# Quality of life, anxiety and cancer worry following hereditary cancer testing: a 6-month Swedish follow-up study

**DOI:** 10.1007/s11136-026-04184-1

**Published:** 2026-02-12

**Authors:** Ylva Heyman, Hanna Röjlar, Carolina Hawranek, Barbro Numan Hellquist, Anna Rosén

**Affiliations:** 1 Department of Diagnostics and Intervention, Oncology, Umeå University, Umeå, Sweden; 2 Division of Oncology, Department of Clinical Sciences Lund, Lund University, Lund, Sweden; 3 Department of Haematology, Oncology and Radiation Physics, Skåne University Hospital, Lund, Sweden

**Keywords:** Genetic testing, Hereditary breast and ovarian cancer, Lynch syndrome, Health-related quality of life, Anxiety, Cancer worry

## Abstract

**Purpose:**

As genetic testing becomes increasingly integrated into routine care, understanding its impact on psychological well-being and health-related quality of life (HRQoL) is essential. This study assessed HRQoL, anxiety and cancer worry following hereditary cancer testing in a Swedish clinical setting and identified predictors of these outcomes.

**Methods:**

Participants were recruited from four outpatient cancer genetics clinics across Sweden. Eligible individuals either carried a pathogenic variant or met clinical criteria for familial breast or colorectal cancer. Questionnaires were completed shortly after receiving test results and again 6 months later. HRQoL was measured using the RAND-36, anxiety using the State-Trait Anxiety Inventory and cancer worry using the Cancer Worry Scale. Outcomes were compared with age- and sex-adjusted Swedish population data. Predictors of outcomes were analysed using multivariable linear regression.

**Results:**

A total of 254 participants completed at least one questionnaire. HRQoL improved across all domains over 6 months, while anxiety and cancer worry declined. Participants without a recent cancer diagnosis had scores close to population norms at both time points. Those diagnosed within the previous year had lower HRQoL and higher anxiety and cancer worry, although they improved over time. Poorer outcomes were linked to a recent cancer diagnosis, being an index case, female sex and younger age, while education level and the test result itself were not associated with worse results.

**Conclusion:**

Genetic testing was not associated with substantial short- or medium-term negative effects on HRQoL, anxiety or cancer worry. Individual risk factors should be considered when offering psychosocial support.

**Plain English summary:**

Genetic testing is becoming more widely used, but earlier research on how testing affects everyday life often involved small groups or older data. Testing is also used in new ways today, which creates a need for updated studies to understand people’s experiences and to see who might need extra support. In this study, we examined quality of life, anxiety and cancer-related worry in people tested for hereditary breast, ovarian or colorectal cancer. They completed questionnaires shortly after receiving their results and again 6 months later. We compared their answers with those from the general Swedish population. Quality of life improved over time, and anxiety and cancer worry decreased. People without a recent cancer diagnosis had scores similar to population norms. Those with a recent diagnosis had lower scores but also improved. Younger people, women and those recently diagnosed with cancer reported more anxiety and worry and lower quality of life scores. Education level and the test result itself did not affect these outcomes. Overall, genetic testing did not appear to cause lasting negative effects. Support may be most helpful for people with these risk factors.

**Supplementary Information:**

The online version contains supplementary material available at 10.1007/s11136-026-04184-1.

## Introduction

Pathogenic variants (PVs) in *BRCA1, BRCA2* and *PALB2*, which cause hereditary breast and ovarian cancer syndrome (HBOC), and in mismatch repair genes associated with Lynch syndrome (*MSH2, MSH6, MLH1, PMS2*), significantly increase cancer risk. Women with HBOC have a 69–72% lifetime risk of breast cancer and a 40% (*BRCA1*) or 17% (*BRCA2*) risk of ovarian cancer by age 80 [1]. Individuals with Lynch syndrome face a 15–46% lifetime risk of colorectal cancer, as well as elevated risks of endometrial, ovarian and other cancers [2].

Genetic testing is offered to individuals with cancer who meet relevant clinical criteria to assess whether their diagnosis is associated with a PV in a cancer predisposition gene. If a PV is identified, at-risk relatives can be offered predictive testing and preventive measures. For both HBOC and Lynch syndrome, risk-reduction strategies include enhanced surveillance (annual breast magnetic resonance imaging or colonoscopy) and/or risk-reducing surgeries, which have been shown to reduce cancer incidence and mortality [3–5]. In families with an aggregation of breast or colorectal cancer but no identified PV, intensified surveillance may still be considered, although its cost-effectiveness is debated [3, 5], and changed criteria in national guidelines have resulted in a decrease of such recommendations. As more individuals live with an identified hereditary cancer risk, understanding their experiences and patient-reported outcomes is essential for developing targeted mental health support and services.

Previous studies on health-related quality of life (HRQoL), anxiety and cancer worry in individuals undergoing genetic testing for hereditary cancer have shown that distress often peaks shortly after test disclosure, with levels generally normalising over time. A systematic review involving 2,442 cancer-unaffected women with HBOC found that distress increased shortly after testing, while HRQoL remained largely unaffected [6]. Similarly, a review of 526 cancer-affected carriers reported early but often transient distress and anxiety [7]. Research on Lynch syndrome is more limited but suggests comparable short-term psychological effects following testing [8]. Individuals with a strong family history but no identified PV may also experience a psychological burden. Some studies report higher distress in those with a positive genetic result compared to those with a negative result but familial cancer risk [9, 10], while others find similar levels of psychological well-being across these groups [11–19].

The limitations of prior studies include having small sample sizes and outdated data that may not reflect current clinical practice, particularly given the widespread use of mainstream genetic testing where non-genetic healthcare professionals offer testing directly to patients. Research is needed to identify specific risk factors to better support individuals at higher risk of psychological distress.

This study evaluates HRQoL, anxiety and cancer worry in individuals undergoing genetic testing for HBOC or Lynch syndrome, both at the time of testing and 6 months afterwards, compared to a normative Swedish reference population. Additionally, it examines potential predictors of these patient-reported outcomes.

## Materials and methods

### Study population

Participants in this study were recruited from outpatient cancer genetics clinics across Sweden as part of the DIRECT study (Clinical Trial Identifier: NCT04197856, www.umu.se/direct) [20]. Recruitment sites included Umeå University Hospital, Karolinska University Hospital, Sahlgrenska University Hospital and Skåne University Hospital. Eligible participants were individuals aged 18 years or older who either carried a PV in a cancer susceptibility gene (*BRCA1, BRCA2, PALB2, MLH1, MSH2, MSH6, PMS2*) or met the clinical and pedigree-based criteria for familial breast or colorectal cancer. Both index cases and individuals undergoing predictive testing were included. Some index cases underwent genetic testing as part of a mainstream testing programme, where testing was integrated into routine cancer care. To be included in the DIRECT study, participants also needed to have at least one at-risk relative who was eligible for genetic counselling within one year of the participant’s genetic test result. For the present analyses, participants were included if they had completed at least one patient-reported outcome measure (RAND-36, STAI or CWS) at baseline and/or follow-up. Further details on the study design, as well as the inclusion and exclusion criteria, are available elsewhere [20, 21].

The study was approved by the Swedish Ethical Review Authority (Dnr 2019–02647) and its subsequent amendments (Dnr 2020–01176, Dnr 2021–00718 and Dnr 2021–05890). All participants provided written and oral informed consent prior to participation.

### Questionnaires

The participants completed questionnaires at two time points. The first round was administered about 0–3 weeks after they received their genetic test results, when they attended a genetic consultation (baseline, 0 months). The second round was distributed approximately 6 months after the first. Questionnaires were distributed either by post or electronically, according to each participant’s preference stated at inclusion. Among those given a choice, 69% preferred paper-based questionnaires. Electronic questionnaires were administered via the Swedish national digital healthcare platform (1177.se). Analog surveys were posted to each respondent’s home address. Non-respondents received one reminder for each questionnaire.

The questionnaires included validated instruments to measure generic HRQoL using the RAND 36-Item Health Survey (RAND-36) [22], anxiety with the State-Trait Anxiety Inventory (STAI) [23] and cancer worry using the Cancer Worry Scale (CWS) [24].

#### Health-related quality of life

The RAND-36 is a widely used instrument for assessing HRQoL, with the Swedish translation demonstrating high reliability and validity [25, 26]. It consists of 36 questions grouped into eight multi-item domains, capturing physical functioning, role limitations caused by physical problems, bodily pain, general health, vitality/energy/fatigue, social functioning, emotional well-being and role limitations caused by emotional problems [22]. Each scale is scored based on specific response options, with scores transformed to a 0 to 100 scale, where 0 represents the worst possible health status and 100 the best. The final score for each domain is obtained by calculating the mean of the scale scores [22].

In addition to the domain scores, two composite scores are calculated: the Physical Component Score (PCS) and the Mental Component Score (MCS). The PCS is based on the mean scores from the following domains: bodily pain, physical functioning, role limitations due to physical health problems and vitality. The MCS is calculated using the mean scores from emotional well-being, social functioning and role limitations due to emotional problems.

#### State and trait anxiety

The STAI is composed of two 20-item subscales: the state subscale (STAI-S), which measures the current level of anxiety, and the trait subscale (STAI-T), which assesses relatively stable personal tendencies to experience anxiety [23]. Each item is rated on a four-point Likert scale, and higher total scores indicate higher levels of anxiety. For each subscale, item scores are summed, giving a total score range of 20 to 80 [23]. The STAI has demonstrated good reliability and validity across research and clinical settings [27], including in Swedish populations [28].

#### Cancer worry

The CWS is an eight-item scale that measures both the frequency and severity of cancer-related worry, along with its impact on mood and daily functioning [24]. Each item is rated on a four-point Likert scale, ranging from “never” to “almost always” for frequency items and “not at all” to “a lot” for severity items [29]. The total score ranges from 8 to 32, with higher scores reflecting greater worry or a stronger impact on daily life. The Swedish translation of the CWS used in this study has previously been applied in a population-based setting, and the full questionnaire is available elsewhere [29]. Previous studies have demonstrated that the CWS has strong validity and reliability in populations with hereditary cancer risk and among cancer survivors [30–32]. A Swedish version has been used in research settings [29], although a psychometric validation has not been published.

### Normative reference groups

Normative data are crucial for assessing whether the scores of a specific group deviate from population averages. In this study, we used normative reference data for the CWS, STAI and RAND-36 from the Swedish population [28, 29, 33] weighted by sex and age group.

HRQoL, assessed using the RAND-36, was compared to a sample of 3,432 individuals from the general population in a Swedish county. These data were collected by mail in 2015 and stratified by sex and age to ensure representativeness [33].

State and trait anxiety scores from our sample, measured using the STAI, were compared to a stratified, randomly selected sample of 180 individuals (90 men and 90 women) from an urban Swedish population, divided into three age groups: 28–45, 48–85 and over 85 years. These data were collected via mailed questionnaires in 1992 [28].

The CWS results in our study were compared to a population-based sample of 943 individuals, aged 18–74, randomly selected from the Swedish Population Register. Data collection took place in 2018 through an electronic survey conducted by our research group and distributed through the Laboratory of Opinion Research at Gothenburg University [29].

### Statistical analyses

Summary statistics were used to describe the overall sample and each genetic test result subgroup, with mean and standard deviation (SD) reported for age and column percentages provided for ordinal and nominal variables.

For multi-item scales, the median was imputed if at least 50% of items were completed; otherwise, the individual was excluded from analysis for that scale, consistent with the half-scale approach described for RAND-36 scoring [34]. A sensitivity analysis was also conducted, including only questionnaires with complete responses. Patient-reported outcomes, including mean scores and SDs, were calculated at baseline and 6 months post-counselling. Comparisons between the baseline and 6-month scores were performed using the Wilcoxon signed-rank test. Analyses were then stratified by recent cancer diagnosis (yes/no), defined as a diagnosis within the same calendar year as testing or the year prior to testing.

Subgroup analyses were conducted for individuals with breast and/or ovarian cancer and those with colorectal cancer. Patient-reported outcomes at baseline and 6 months post-counselling were stratified by genetic test result (positive = PV; negative = no PV). Differences in mean scores between subgroups were assessed using the Mann–Whitney U test.

Potential predictors of cancer worry, state and trait anxiety and HRQoL (measured by physical and MCSs) at baseline were analysed using multivariable linear regression models. Positively skewed scales (STAI-T and CWS) were log-transformed, while negatively skewed scales (PCS, MCS and STAI-S) underwent reflected log transformation, ensuring that all scores met linear regression assumptions. Since the indications for testing and family cancer risk group were highly correlated, two separate multivariable models were created for each outcome to avoid multicollinearity. One model included indications for testing sex, age (< 40, 40–60, ≥ 61) and education, while the other included family cancer risk group, recent cancer diagnosis (yes/no), sex, age (< 40, 40–60, ≥ 61) and education. The same analyses were performed for outcomes at 6 months post-counselling.

All analyses were conducted with an alpha level set at 0.05. Statistical analyses were performed using SPSS (version 28.0, IBM Corp, Armonk, NY) and R (version 4.4.0) [35].

## Results

### Participant characteristics

Of the 273 individuals who fulfilled the eligibility criteria and accepted participation, 254 completed at least one questionnaire and were included in the analyses. The response rate was 94% at baseline and 87% at follow-up. Of the 254 participants, 56% had HBOC and 17% had Lynch syndrome. Additionally, 19% had a negative test but were classified as having familial breast cancer, and 7% as having familial colorectal cancer. The mean age at testing was 57 years (SD = 13), with no substantial age differences between subgroups. Most participants were female (76%), and 36% had a university or college degree (Table 1).

**Table 1:** Baseline characteristics by positive test (pathogenic variant) and negative test (familial cancer)

			Positive test		Negative test		Total (n = 254)
			HBOC (n = 143)	Lynch syndrome (n = 43)	Familial breast cancer (n = 49)	Familial colorectal cancer (n = 19)	
Mean (SD) age at testing			57 (14)	57 (15)	56 (13)	62 (8)	57 (13)
Age group	≤ 40		19 (13.3)	7 (16.3)	6 (12.2)	0 (0.0)	32 (12.6)
	40–60		64 (44.8)	15 (34.9)	23 (46.9)	8 (44.4)	110 (43.5)
	≥ 61		60 (42.0)	21 (48.8)	20 (40.8)	10 (55.6)	111 (43.9)
Sex	Female		114 (79.7)	23 (53.5)	49 (100.0)	7 (36.8)	193 (76.0)
	Male		29 (20.3)	20 (46.5)	0 (0.0)	12 (63.2)	61 (24.0)
Education	Less than 9 years of schooling		5 (3.5)	3 (7.0)	2 (4.1)	0 (0.0)	10 (3.9)
	Completed mandatory school or equivalent (9 years)		22 (15.4)	4 (9.3)	2 (4.1)	5 (26.3)	33 (13.0)
	Completed high school or equivalent (12 years)		26 (18.2)	6 (14.0)	6 (12.2)	6 (31.6)	44 (17.3)
	Education of at least 1 year beyond high school		31 (21.7)	7 (16.3)	10 (20.4)	6 (31.6)	54 (21.3)
	University or college degree		51 (35.7)	16 (37.2)	24 (49.0)	1 (5.3)	92 (36.2)
	Missing		8 (5.6)	7 (16.3)	5 (10.2)	1 (5.3)	21 (8.3)
Cancer diagnosis	Cancer	Yes	97 (67.8)	31 (72.1)	48 (98.0)	15 (83.3)	191 (75.5)
		No	39 (27.3)	10 (23.3)	0 (0.0)	0 (0.0)	49 (19.4)
	Breast cancer	Yes	69 (48.3)	1 (2.3)	48 (98.0)	0 (0.0)	118 (46.6)
		No	67 (46.9)	40 (93.0)	0 (0.0)	15 (83.3)	122 (48.2)
	Ovarian cancer	Yes	23 (16.1)	1 (2.3)	0 (0.0)	0 (0.0)	24 (9.5)
		No	113 (79.0)	39 (92.9)	48 (98.0)	15 (83.3)	216 (85.4)
	Colorectal cancer ^a^	Yes	4 (2.8)	24 (55.8)	1 (2.0)	15 (83.3)	44 (17.4)
		No	132 (92.3)	17 (39.5)	47 (95.9)	0 (0.0)	196 (77.5)
	Other cancer/cancers	Yes	8 (5.6)	13 (30.2)	1 (2.0)	3 (16.7)	25 (9.9)
		No	128 (89.5)	28 (65.1)	47 (95.9)	12 (66.7)	215 (85.0)
	Missing		7 (4.9)	2 (4.7)	1 (2.0)	3 (16.7)	13 (5.1)
Reason for testing	Index case testing		83 (58.0)	30 (69.8)	49 (100.0)	18 (94.7)	180 (70.9)
	Predictive test		59 (41.3)	13 (30.2)	0 (0.0)	0 (0.0)	72 (28.3)
	Missing		1 (0.7)	0 (0.0)	0 (0.0)	1 (5.3)	2 (0.8)
Site	Umeå		64 (44.8)	19 (44.2)	20 (40.8)	13 (68.4)	116 (45.7)
	Göteborg		27 (18.9)	12 (27.9)	28 (57.1)	4 (21.1)	71 (28.0)
	Lund		46 (32.2)	10 (23.3)	0 (0.0)	0 (0.0)	56 (22.0)
	Stockholm		6 (4.2)	2 (4.7)	1 (2.0)	2 (10.5)	11 (4.3)

A majority (n = 180, 71%) were index patients who underwent genetic testing following a cancer diagnosis. The remaining participants were tested through predictive testing as at-risk relatives. In total, 76% of all participants had a current or previous cancer diagnosis, with breast cancer being the most common (47%) (Table 1). Among all participants, 50% had been diagnosed with cancer in the same year or the year before testing, with rates varying across subgroups: 50% in the HBOC group, 49% in the Lynch syndrome group, 61% in the familial breast cancer group and 32% in the familial colorectal cancer group. Cross-tabulations of testing indication, genetic test result and cancer diagnosis, including timing in relation to testing, are presented in Supplementary Table S1.^1^

### Longitudinal patient-reported outcomes

**Fig. 1:**
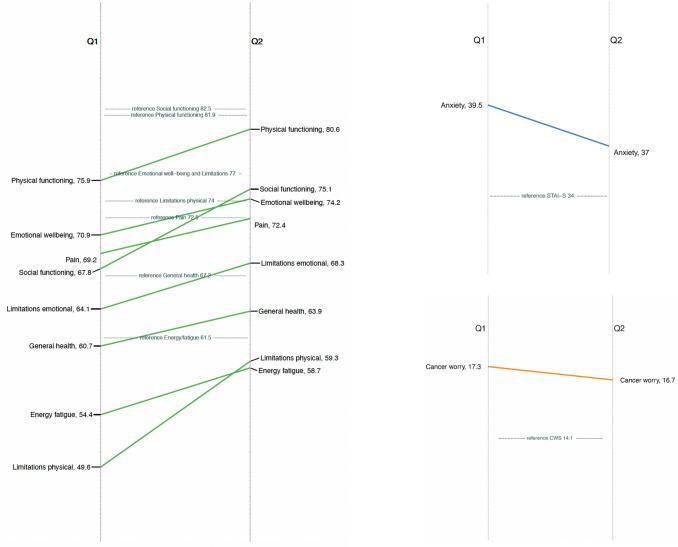
Changes in health-related quality of life, anxiety and cancer worry from baseline to 6-month follow-up. The figure shows mean scores for RAND-36 domains (green), STAI-S anxiety (blue) and Cancer Worry Scale (red) at baseline (Q1) and 6-month follow-up (Q2) among individuals undergoing genetic testing for hereditary breast and ovarian cancer or Lynch syndrome. The central grey bars represent Swedish population reference values. Normative datasets for RAND-36, STAI-S and Cancer Worry Scale were weighted by sex and age group using stratified data.

HRQoL improved across all domains from baseline (shortly after genetic testing and counselling) to 6 months post-counselling. In domains such as physical functioning, emotional well-being, pain and energy/fatigue, scores approached population norms. Other domains remained below normative levels after 6 months (Fig. 1). These changes were statistically significant in all domains except role limitations due to emotional problems and bodily pain (Supplementary Table S2). Cancer worry and state anxiety also decreased significantly, though both remained elevated compared to population norms at follow-up (Fig. 1, Supplementary Table S2). Similar results were observed in the sensitivity analysis including only questionnaires with complete responses (Supplementary Table S3).

Stratified analyses showed that participants diagnosed with cancer within the past year had lower HRQoL and higher anxiety and cancer worry than those without a recent diagnosis (Supplementary Figure S1 and Table S2). Among those with a recent cancer diagnosis, HRQoL improved significantly in all domains except role limitations due to emotional problems. In contrast, no significant HRQoL changes were observed in participants without a recent diagnosis. State anxiety declined significantly in both groups, while reductions in trait anxiety and cancer worry were only seen in those without a recent diagnosis. Compared to population norms, participants with recent cancer consistently reported lower HRQoL and higher anxiety and cancer worry at both time points. In contrast, participants without recent cancer generally reported HRQoL at or above population norms, except for lower scores in social functioning and role limitations due to physical health. Their energy/fatigue scores were higher than the norm at both time points. Anxiety levels were elevated at baseline but approached normative values after 6 months, and cancer worry had nearly normalised by that point (Supplementary Figure S1).

Subgroup analyses of individuals with breast and/or ovarian cancer or colorectal cancer showed similar results for those with and without a positive genetic test, both at baseline and at 6 months. One exception was lower PCS at baseline in individuals with a positive HBOC result (Supplementary Table S4–S5).

### Risk and protective factors

**Fig. 2:**
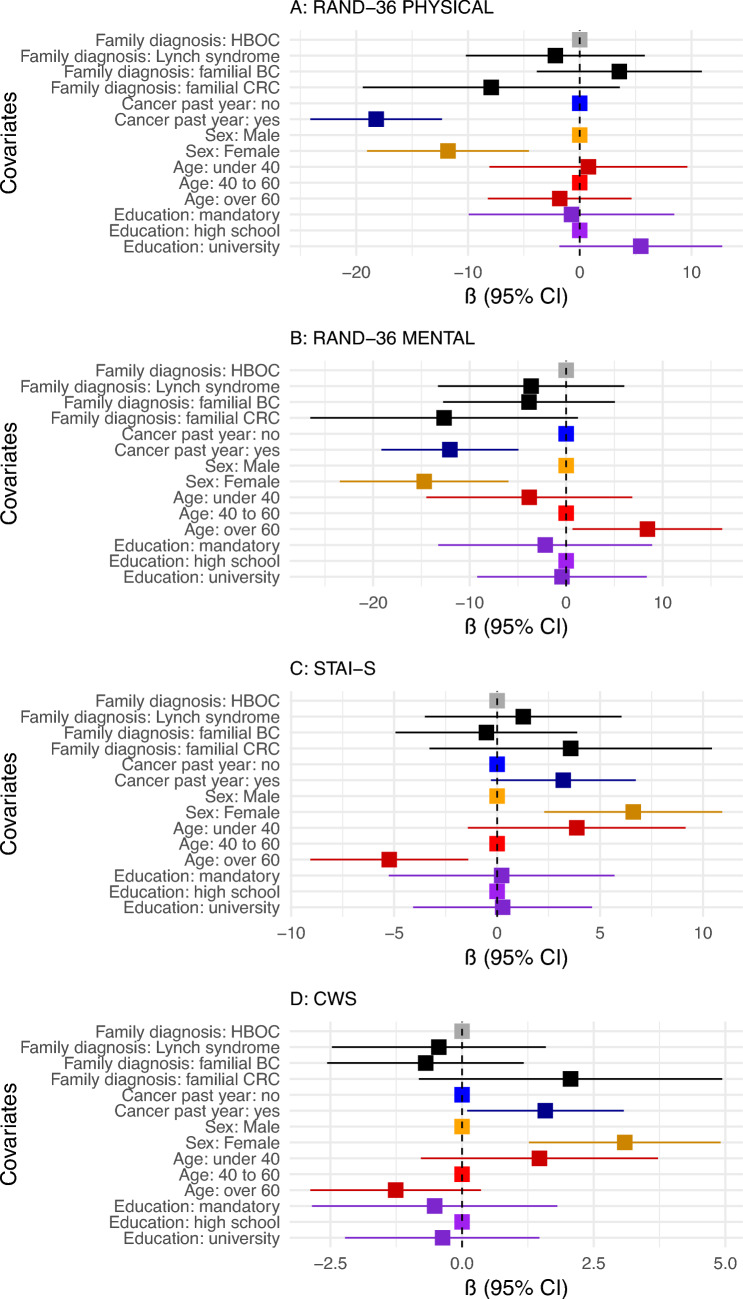
Predictors of health-related quality of life, anxiety and cancer worry. The forest plot presents multivariable linear regression results for predictors of physical and mental health-related quality of life (RAND-36), state anxiety (STAI-S) and cancer worry (Cancer Worry Scale). These outcomes were measured at baseline, shortly after testing and counselling, among individuals tested for hereditary breast and ovarian cancer syndrome or Lynch syndrome. Adjusted β coefficients (95% confidence intervals) represent the strength and direction of associations

Several factors influenced HRQoL, anxiety and cancer worry at baseline (shortly after testing and counselling) (Fig. 2 and Supplementary Table S6) and at 6 months (Supplementary Table S7). However, the type of genetic finding (HBOC, Lynch syndrome, familial breast cancer or familial colorectal cancer) was not associated with significant differences in these outcomes at either time point, after adjusting for recent cancer diagnosis, sex, age and education (Fig. 2).

Undergoing genetic testing as an index case was consistently associated with poorer outcomes than predictive testing. At baseline, index cases had significantly lower PCS (adjusted β = − 18.11, 95% CI − 24.45 to − 11.77) and MCS (adjusted β = − 15.65, 95% CI − 23.13 to − 8.16). These differences were less pronounced at 6 months (PCS: β = − 8.52, 95% CI − 15.28 to − 1.76; MCS: β = − 10.81, 95% CI − 18.61 to − 3.01). Cancer worry was higher for index cases at both time points (baseline: β = 2.73, 95% CI 1.17 to 4.28; 6 months: β = 2.62, 95% CI 0.97 to 4.27), while state anxiety remained unchanged.

A recent cancer diagnosis (within the current or past year) was associated with lower HRQoL at baseline (PCS: β = − 18.22, 95% CI − 24.11 to − 12.33; MCS: β = − 12.05, 95% CI − 19.15 to − 4.95), higher cancer worry (β = 1.58, 95% CI 0.10 to 3.07) and higher but not statistically significant state anxiety (β = 3.21, 95% CI − 0.30 to 6.73). The impact remained at 6 months.

Female sex was associated with lower HRQoL at baseline (PCS: β = − 8.32, 95% CI − 15.09 to − 1.55; MCS: β = − 11.68, 95% CI − 19.68 to − 3.68), higher state anxiety (β = 6.03, 95% CI 2.07 to 10.00) and higher cancer worry (β = 2.38, 95% CI 0.72 to 4.05). These differences were slightly reduced at 6 months.

Older age (≥ 61) was associated with higher MCS (β = 9.53, 95% CI 2.16 to 16.90) and lower state anxiety at baseline (β = − 6.41, 95% CI − 10.06 to − 2.76). The association with state anxiety remained significant at 6 months, along with slightly lower cancer worry (β = − 1.65, 95% CI − 3.24 to − 0.06). Education level did not significantly affect HRQoL, anxiety or cancer worry at either time point.

## Discussion

This study assessed patient-reported outcomes in a Swedish cohort following genetic testing for HBOC or Lynch syndrome. Over 6 months, HRQoL improved across all domains. Among participants without a cancer diagnosis in the past year, HRQoL approached population norms, while those with a recent cancer diagnosis had persistently lower HRQoL across all domains. Cancer worry and anxiety also declined during the follow-up period. For participants without a recent cancer diagnosis, levels neared population norms, whereas they remained elevated among those with cancer. A recent cancer diagnosis, being an index case and female sex were associated with lower mental and physical HRQoL and higher cancer worry at both baseline and follow-up. Female sex was also associated with higher anxiety, while older age was linked to lower anxiety at both time points.

Genetic testing for HBOC has been available since the 1990s, and testing for Lynch syndrome was introduced in the early 2000s. Initially, there were widespread concerns about the potential negative impact of genetic testing on mental health and well-being. However, previous studies, similar to our findings, show that while genetic testing may cause temporary distress, long-term effects on mental health and HRQoL are generally limited [6–8]. The initial distress may be linked to challenging decisions following test results, such as discussing inherited cancer risk with family members or considering risk-reducing interventions. For index cases, genetic testing often coincides with a recent cancer diagnosis, which itself contributes to increased anxiety and reduced HRQoL, as reflected in our results. Over time, many individuals seem to adapt, leading to a decline in distress.

Identifying factors associated with poorer outcomes is crucial for providing targeted support. While patient-reported outcomes generally improve at the group level, some individuals may experience long-term distress, possibly due to underlying risk factors. Consistent with our findings, previous studies of HBOC and Lynch syndrome have highlighted several risk factors, including a cancer diagnosis [12, 36], younger age (particularly under 40) [37] and female sex [12]. These factors have been associated with lower HRQoL and elevated cancer worry. Additional risk factors for a decline in HRQoL, anxiety and cancer worry after genetic testing include high distress prior to testing, a history of depression [14, 36, 38], having young children [36, 38], having lost a relative to cancer [38, 39] and using passive or avoidant coping styles [39].

A positive genetic test result for a PV was not associated with worse patient-reported outcomes compared to a negative result in our cohort. All individuals with negative results were index cases, with a prior cancer diagnosis, who sought genetic counselling due to a family history of cancer. In subgroup analyses of individuals with a personal history of breast/ovarian or colorectal cancer, we found no significant differences in HRQoL, anxiety or cancer worry between those with positive and negative results. These findings are consistent with previous studies, which report that patients undergoing mainstream genetic testing during cancer care have similar patient-reported outcomes to other cancer patients [18, 40]. For example, an Australian study found that cancer-specific distress levels in women with breast cancer were comparable regardless of their genetic results [15].

Research on predictive genetic testing in cancer-free individuals, however, has yielded mixed results. Some studies report short-term distress following a positive result [9, 10], while others show no significant differences in psychological well-being between those with positive and negative results, either in the short term [13, 16] or after follow-ups of up to three years [17, 19] or five years [38]. Interestingly, a negative genetic test result can also cause distress. One study found higher anxiety and depression in women with a negative result for HBOC or Lynch syndrome compared to those with a positive result [41]. A qualitative study of women testing negative for HBOC highlighted conflicting emotions, including relief, happiness, guilt, fear and anger [42]. Taken together, these findings suggest that the impact on psychological and physical well-being may stem more from the testing process itself or a recent cancer diagnosis than from the genetic test result.

This study has several strengths. To our knowledge, it is the first to evaluate patient-reported outcomes in a contemporary Swedish population with HBOC and Lynch syndrome, which is particularly relevant given the recent implementation of mainstream genetic testing programmes. Recruitment from multiple centres across Sweden contributed to a diverse participant pool, and high response rates at baseline (94%) and follow-up (87%) support the representativeness of the findings. The inclusion of men with PVs in *BRCA1* and *BRCA2* and individuals with Lynch syndrome, groups often underrepresented in similar studies, further strengthens the study.

However, several limitations should be acknowledged. As with all self-reported data, the use of patient-reported outcomes may have introduced response bias. Still, the validated instruments used in this study have shown reliability in similar populations [26, 27, 31, 32]. The Swedish normative data for the STAI are from the 1990s, which may limit their relevance for current comparisons. In contrast, the normative data for RAND-36 and the CWS are based on more recent Swedish populations, increasing their applicability. Another limitation is the lack of pre-test data on HRQoL, anxiety or cancer worry, which restricts the ability to assess changes specifically related to genetic testing. Furthermore, the 6-month follow-up may not reflect longer-term outcomes. Still, evaluating outcomes shortly after testing and again at 6 months offers valuable insights into short- and medium-term trends during the initial adjustment period. Finally, as the study was conducted in Sweden, within a publicly funded healthcare system, the findings should be interpreted in this context and may not be fully generalisable to settings with unequal or limited access to healthcare.

## Conclusion

In this Swedish cohort of individuals tested for HBOC and Lynch syndrome, HRQoL improved over the 6 months following post-test counselling, and both cancer worry and anxiety declined. Among those without a recent cancer diagnosis, patient-reported outcomes were generally at or near population norms at both time points, suggesting limited changes following testing. In contrast, individuals diagnosed with cancer within the past year consistently reported lower HRQoL and elevated anxiety and cancer worry. Poorer outcomes were associated with a recent cancer diagnosis, being an index case (often overlapping), female sex and younger age. These findings suggest that genetic testing does not have a substantial long-term negative impact on quality of life, anxiety or cancer worry, and they highlight the importance of tailored psychosocial support in hereditary cancer care.

## Supplementary Information

Below is the link to the electronic supplementary material.

Supplementary file1 (DOCX 1090 KB)

## Data Availability

Deidentified data may be available to qualified researchers upon reasonable request to the corresponding author, subject to ethical approval and data use agreements. Due to privacy regulations, individual-level data cannot be shared publicly.

## Footnotes

1 ^a^One case of colon cancer in situ and one case of appendix cancer are coded as colorectalcancer All data are presented as counts with column percentages in parentheses, unless otherwisespecified HBOC = Hereditary Breast and Ovarian Cancer
