# Supplementary material for: Quality of life, anxiety and cancer worry following hereditary cancer testing: a 6-month Swedish follow-up study
Source: Qual Life Res. 2026 Feb 12;35(3):70. doi: 10.1007/s11136-026-04184-1 (PMC12901253; doi:10.1007/s11136-026-04184-1)
Supplement: Supplementary file 1 — Supplementary file1 (DOCX 1090 KB) [file 11136_2026_4184_MOESM1_ESM.docx]

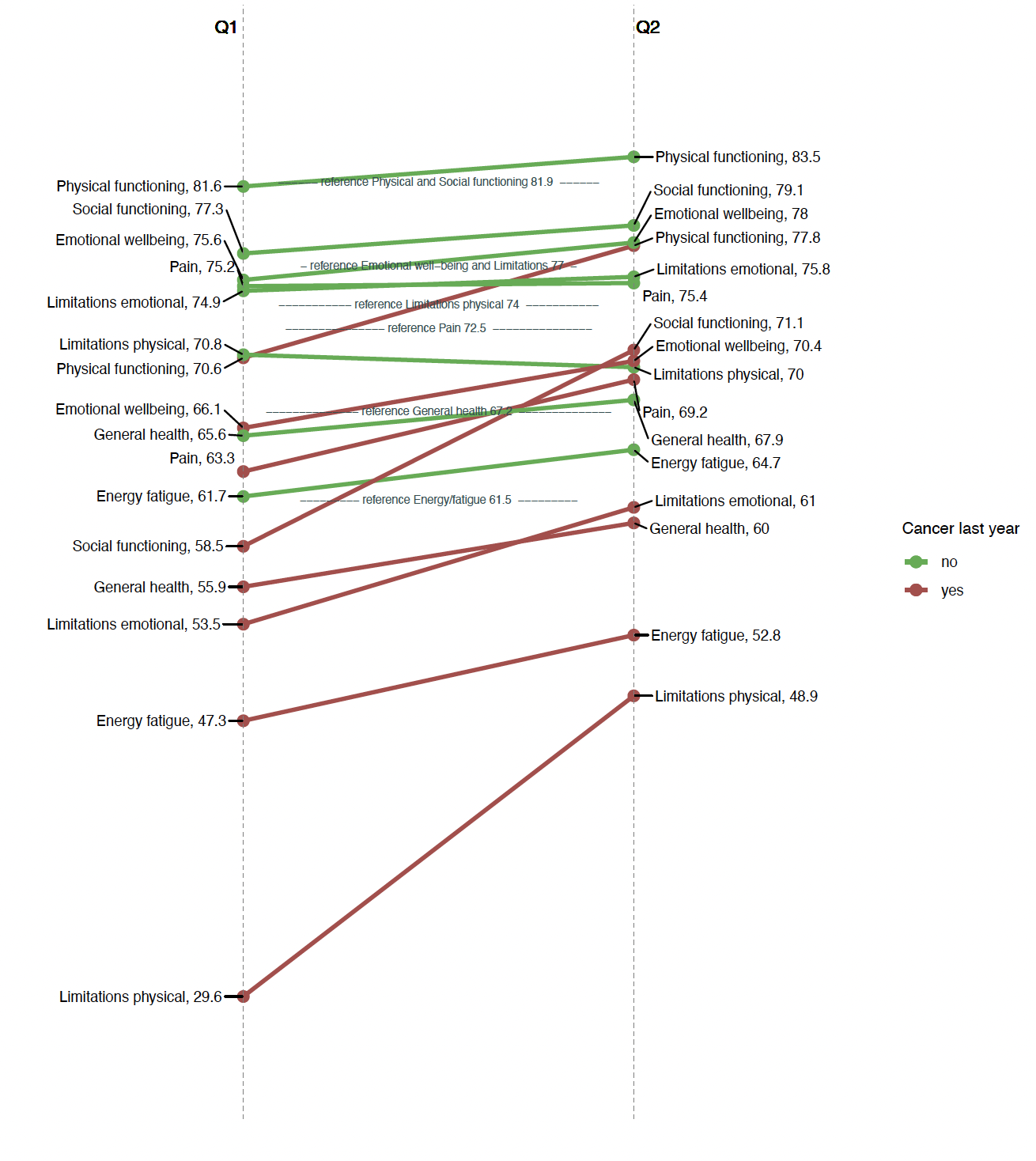


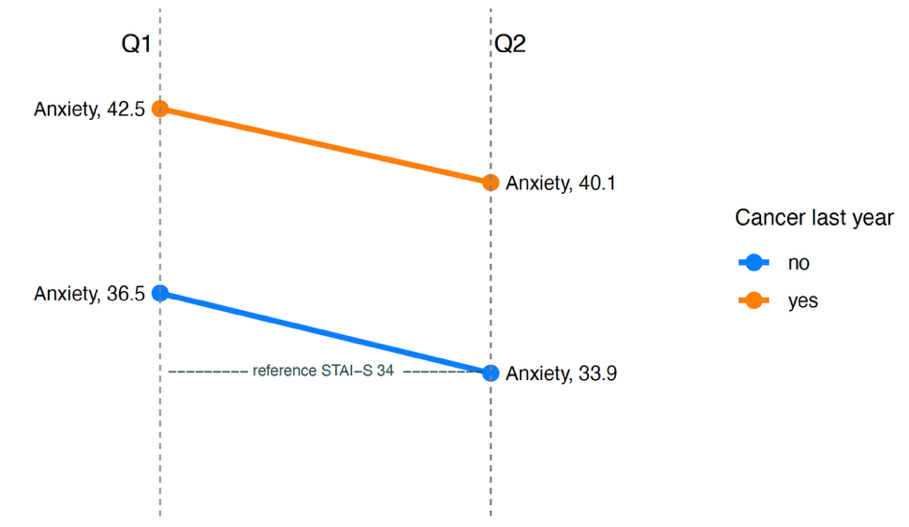


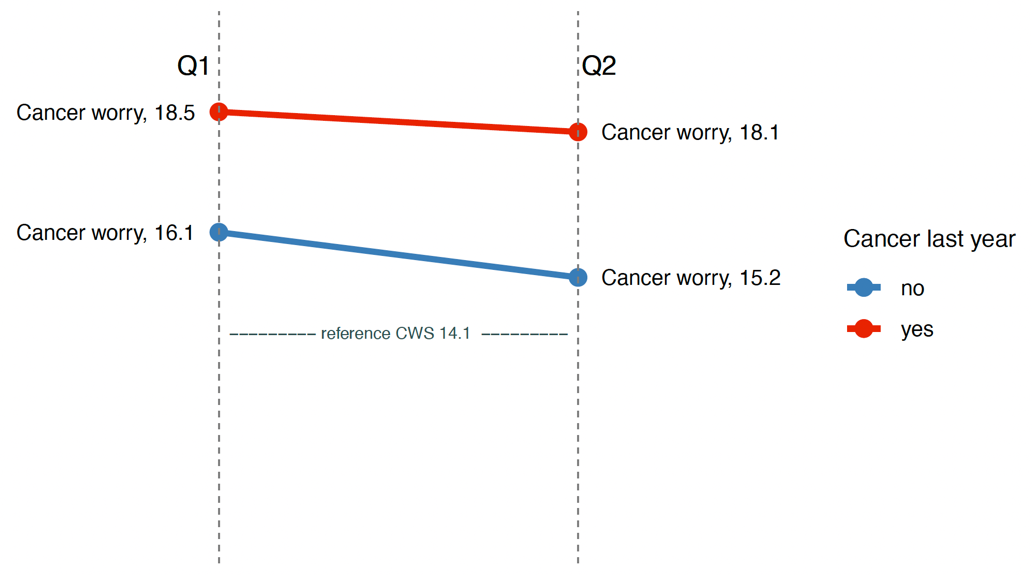


**Supplementary Figure S1. Changes in health-related quality of life, anxiety and cancer worry from baseline to six-month follow-up, stratified by recent cancer diagnosis.**
The figure shows mean scores for RAND-36 domains, STAI-S anxiety scores and Cancer Worry Scale scores at baseline (Q1) and six-month follow-up (Q2) among individuals undergoing genetic testing for hereditary breast and ovarian cancer syndrome or Lynch syndrome, stratified by recent cancer diagnosis (during the current or previous year) or not. The central grey bars represent Swedish population reference values. Normative datasets for RAND-36, STAI-S and Cancer Worry Scale were weighted by sex and age group.

Supplementary Table S1. Crosstabulation of indication for testing and related characteristics

|  | | | Indication for testing | | |  |
| --- | --- | --- | --- | --- | --- | --- |
|  |  |  | Index case testing^a^ | Predictive testing | Missing | Total |
| Family diagnosis | Positive test | HBOC | 83 (46.6) | 59 (81.9) | 1 (50.0) | 143 (56.3) |
|  |  | Lynch syndrome | 29 (16.3) | 13 (18.1) | 0 (0.0) | 43 (16.9) |
|  |  |  |  |  |  |  |
|  | Negative test | Familial breast cancer | 49 (27.5) | 0 (0.0) | 0 (0.0) | 49 (19.3) |
|  |  | Familial colorectal cancer | 17 (9.6) | 0 (0.0) | 1 (50.0) | 19 (7.5) |
|  |  |  |  |  |  |  |
| Cancer diagnosis |  | Yes | 175 (97.8) | 16 (22.2) | 1 (50.0) | 191 (75.5) |
|  |  | No | 0 (0.0) | 48 (66.7) | 0 (0.0) | 49 (19.4) |
|  |  | Missing | 4 (2.2) | 8 (11.1) | 1 (50.0) | 13 (5.1) |
|  |  |  |  |  |  |  |
| Cancer diagnosis in the past year |  | Yes | 122 (67.8) | 6 (8.3) | 0 (0.0) | 128 (50.4) |
|  |  | No | 58 (32.2) | 66 (91.7) | 1 (50.0) | 125 (49.2) |
|  |  | Missing | 0 (0.0) | 0 (0.0) | 1 (50.0) | 1 (0.4) |

^a^The first individual in a family to undergo genetic testing. Some index cases were tested as part of the mainstream testing program.

All data are presented as counts with column percentages in parentheses.

HBOC = Hereditary Breast and Ovarian Cancer

Supplementary Table S2. Comparison of quality of life measures, anxiety and cancer worry at baseline and 6 months, stratified by cancer diagnosis in the past year

|  | All | | |  | Cancer diagnosis in the past year of testing^a^ | | |  | No cancer diagnosis in the past year of testing | | |
| --- | --- | --- | --- | --- | --- | --- | --- | --- | --- | --- | --- |
|  | Baseline^b^ | 6 months | Test (P-value)^c^ |  | Baseline^b^ | 6 months | Test (P-value)^c^ |  | Baseline^b^ | 6 months | Test (P-value)^c^ |
|  | Mean (SD) | Mean (SD) | P-value |  | Mean (SD) | Mean (SD) | Mean (SD) |  | Mean (SD) | Mean (SD) | Mean (SD) |
| Physical functioning | 75.9 (24.4) | 80.6 (20.4) | **<0.01** |  | 70.6 (25.7) | 77.8 (20.2) | **<0.01** |  | 81.6 (21.8) | 83.5 (20.3) | 0.56 |
| Role limitations due to physical health problems | 49.6 (44.4) | 59.3 (41.7) | **0.01** |  | 29.6 (38.9) | 48.9 (41.6) | **<0.01** |  | 70.8 (40.1) | 70.0 (39.4) | 0.29 |
| Role limitations due to personal/emotional problems | 64.1 (42.8) | 68.3 (40.2) | 0.48 |  | 53.5 (43.7) | 61.0 (41.9) | 0.19 |  | 74.9 (39.3) | 75.8 (37.4) | 0.56 |
| Energy/vitality | 54.4 (23.5) | 58.7 (23.9) | **0.01** |  | 47.3 (21.4) | 52.8 (22.2) | **0.01** |  | 61.7 (23.5) | 64.7 (24.3) | 0.31 |
| Emotional wellbeing | 70.9 (20.3) | 74.2 (18.8) | **0.04** |  | 66.1 (19.6) | 70.4 (18.2) | **0.04** |  | 75.6 (19.9) | 78.0 (18.7) | 0.29 |
| Social functioning | 67.8 (29.3) | 75.1 (25.3) | **<0.01** |  | 58.5 (28.3) | 71.1 (24.7) | **<0.01** |  | 77.3 (27.5) | 79.1 (25.5) | 0.86 |
| Bodily pain | 69.2 (27.1) | 72.4 (25.7) | 0.10 |  | 63.3 (25.3) | 69.2 (25.3) | **0.01** |  | 75.2 (27.8) | 75.4 (25.9) | 0.33 |
| General health | 60.7 (20.7) | 63.9 (20.7) | **0.4** |  | 55.9 (19.4) | 60.0 (20.0) | **0.40** |  | 65.6 (21.0) | 67.9 (20.9) | 0.35 |
| Physical component score | 61.9 (22.8) | 67.0 (21.6) | **<0.01** |  | 53.3 (19.6) | 61.7 (20.2) | **<0.01** |  | 71.0 (22.5) | 72.3 (21.9) | 0.89 |
| Mental component score | 67.6 (27.3) | 72.5 (24.7) | **0.02** |  | 59.4 (26.1) | 67.5 (24.8) | **<0.01** |  | 75.9 (25.9) | 77.7 (23.8) | 0.83 |
|  |  |  |  |  |  |  |  |  |  |  |  |
| State anxiety | 39.5 (13.3) | 37.0 (12.3) | **<0.01** |  | 42.5 (12.8) | 40.1 (12.4) | **0.04** |  | 36.5 (13.0) | 33.9 (11.5) | **0.05** |
| Trait anxiety | 38.1 (11.2) | 36.7 (11.3) | 0.06 |  | 40.2 (11.1) | 39.5 (11.3) | 0.50 |  | 36.1 (10.8) | 33.9 (10.6) | **0.03** |
|  |  |  |  |  |  |  |  |  |  |  |  |
| Cancer worry | 17.3 (5.5) | 16.7 (5.4) | **<0.01** |  | 18.5 (5.5) | 18.1 (5.6) | 0.34 |  | 16.1 (5.2) | 15.2 (4.9) | **<0.01** |

SD=standard deviation

^a^Cancer diagnosis the same year or the year preceding testing.

^b^Questionnaires were completed after genetic testing and counselling (baseline) and again 6 months later.

^c^Differences between means at baseline and 6 months after baseline were calculated using the Wilcoxon signed-rank test.

Supplementary Table S3. Supplementary Table S3. Sensitivity analysis of quality of life, anxiety and cancer worry at baseline and 6 months

|  | Sensitivity analysis including only questionnaires with complete item responses^a^ | | |  |
| --- | --- | --- | --- | --- |
|  | Baseline^b^ | 6 months | Test (P-value)^c^ |  |
|  | Mean (SD) | Mean (SD) | Mean (SD) |  |
| Physical functioning | 76.7 (23.7) | 81.7 (19.8) | **0.04** |  |
| Role limitations due to physical health problems | 50.9 (44.4) | 61.1 (40.9) | **0.02** |  |
| Role limitations due to personal/emotional problems | 64.3 (43.3) | 69.5 (39.5) | 0.34 |  |
| Energy/vitality | 54.9 (23.4) | 60.4 (23.4) | **0.02** |  |
| Emotional wellbeing | 71.0 (20.5) | 74.2 (18.9) | 0.16 |  |
| Social functioning | 68.1 (29.6) | 75.8 (25.0) | **0.01** |  |
| Bodily pain | 70.6 (26.3) | 74.0 (24.7) | 0.23 |  |
| General health | 60.8 (21.0) | 64.6 (20.7) | 0.08 |  |
| Physical component score | 62.8 (22.5) | 68.3 (20.8) | **0.01** |  |
| Mental component score | 67.8 (27.7) | 73.2 (24.3) | 0.07 |  |
|  |  |  |  |  |
| State anxiety | 39.5 (13.2) | 37.0 (12.4) | 0.05 |  |
| Trait anxiety | 38.1 (11.2) | 36.8 (11.3) | 0.07 |  |
|  |  |  |  |  |
| Cancer worry | 17.2 (5.5) | 16.5 (5.5) | 0.18 |  |

SD=standard deviation

^a^Participants were excluded if they had any missing item responses within the scale analysed. This included missing responses to RAND-36 items for RAND-36–based outcomes, STAI items for STAI-S and STAI-T, and CWS items for CWS.

^b^Questionnaires were completed after genetic testing and counselling (baseline) and again 6 months later.

^c^Differences between means at baseline and 6 months after baseline were calculated using the Wilcoxon signed-rank test.

Supplementary Table S4. Comparison of quality-of-life measures, anxiety and cancer worry at baseline and six months in individuals with a breast and/or ovarian cancer diagnosis, stratified by genetic test result (positive = pathogenic variant in *BRCA1, BRCA2* or *PALB2*; negative = no pathogenic variant)

|  | Baseline | | |  | 6 months | | |
| --- | --- | --- | --- | --- | --- | --- | --- |
|  | Positive genetic test (n=87) | Negative genetic test (n=48) | Test (P-value)^a^ |  | Positive genetic test (n=87) | Negative genetic test (n=48) | Test (P-value)^a^ |
|  | Mean (SD) | Mean (SD) |  |  | Mean (SD) | Mean (SD) |  |
| Physical functioning | 68.0 (26.9) | 78.9 (22.3) | **0.02** |  | 74.7 (22.3) | 84.1 (18.3) | **0.04** |
| Role limitations due to physical health problems | 30.8 (40.1) | 47.1 (41.8) | **0.04** |  | 49.4 (42.8) | 61.5 (38.8) | 0.15 |
| Role limitations due to personal/emotional problems | 59.1 (45.6) | 48.5 (41.6) | 0.19 |  | 65.8 (42.3) | 59.8 (39.8) | 0.36 |
| Energy/vitality | 48.0 (22.7) | 52.4 (22.8) | 0.30 |  | 53.8 (24.2) | 56.4 (21.3) | 0.43 |
| Emotional wellbeing | 67.7 (21.1) | 69.7 (20.1) | 0.70 |  | 72.2 (19.7) | 70.0 (17.6) | 0.45 |
| Social functioning | 60.8 (30.3) | 66.4 (29.1) | 0.33 |  | 72.4 (26.1) | 72.8 (22.3) | 0.87 |
| Bodily pain | 62.3 (26.4) | 67.4 (27.5) | 0.29 |  | 69.1 (26.5) | 74.9 (22.3) | 0.30 |
| General health | 56.9 (20.0) | 58.6 (20.4) | 0.53 |  | 60.9 (21.5) | 63.3 (21.2) | 0.49 |
| Physical component score | 53.2 (21.5) | 60.9 (22.4) | **0.04** |  | 61.6 (22.7) | 68.1 (18.9) | 0.14 |
| Mental component score | 62.5 (28.8) | 61.5 (27.4) | 0.82 |  | 70.1 (25.5) | 67.5 (24.0) | 0.44 |
|  |  |  |  |  |  |  |  |
| State anxiety | 41.9 (14.3) | 40.7 (12.9) | 0.81 |  | 38.4 (12.7) | 40.0 (11.8) | 0.41 |
| Trait anxiety | 39.6 (11.7) | 40.2 (11.0) | 0.73 |  | 37.7 (12.0) | 40.1 (11.0) | 0.18 |
|  |  |  |  |  |  |  |  |
| Cancer worry | 18.5 (5.9) | 17.7 (4.9) | 0.50 |  | 17.6 (5.7) | 17.2 (4.6) | 0.73 |

^c^Statistical differences between means were tested using the Mann-Whitney U test

SD=standard deviation

Supplementary Table S5. Comparison of quality of life measures, anxiety and cancer worry at baseline and 6 months in individuals with a colorectal cancer diagnosis, stratified by genetic test result (positive = pathogenic variant in mismatch repair genes; negative = no pathogenic variant)

|  | Baseline | | |  | 6 months | | |
| --- | --- | --- | --- | --- | --- | --- | --- |
|  | Positive genetic test (n=23) | Negative genetic test (n=15) | Test (P-value)^a^ |  | Positive genetic test (n=23) | Negative genetic test (n=15) | Test (P-value)^a^ |
|  | Mean (SD) | Mean (SD) |  |  | Mean (SD) | Mean (SD) |  |
| Physical functioning | 81.1 (18.6) | 70.0 (27.2) | 0.23 |  | 84.3 (18.4) | 81.5 (20.7) | 0.60 |
| Role limitations due to physical health problems | 48.7 (44.5) | 51.8 (48.5) | 0.87 |  | 52.5 (42.1) | 61.5 (47.5) | 0.60 |
| Role limitations due to personal/emotional problems | 68.4 (36.0) | 57.1 (46.1) | 0.58 |  | 66.7 (40.5) | 59.0 (45.5) | 0.70 |
| Energy/vitality | 55.0 (18.9) | 53.0 (20.5) | 0.82 |  | 63.0 (20.5) | 54.2 (25.7) | 0.35 |
| Emotional wellbeing | 70.7 (18.6) | 70.0 (17.9) | 0.84 |  | 75.6 (17.0) | 68.6 (25.3) | 0.60 |
| Social functioning | 69.7 (24.4) | 67.9 (27.6) | 0.90 |  | 74.4 (24.2) | 69.2 (34.1) | 0.76 |
| Bodily pain | 74.1 (24.4) | 64.1 (28.8) | 0.36 |  | 69.4 (28.5) | 66.0 (28.1) | 0.68 |
| General health | 59.0 (17.8) | 60.4 (25.4) | 1.00 |  | 63.3 (19.8) | 63.5 (27.3) | 0.87 |
| Physical component score | 63.6 (18.6) | 59.9 (24.3) | 0.68 |  | 66.5 (21.5) | 65.4 (26.7) | 0.93 |
| Mental component score | 69.6 (20.9) | 65.0 (26.0) | 0.65 |  | 72.2 (23.6) | 65.6 (31.7) | 0.73 |
|  |  |  |  |  |  |  |  |
| State anxiety | 38.3 (12.1) | 39.2 (13.2) | 0.97 |  | 35.5 (11.2) | 36.9 (14.0) | 0.93 |
| Trait anxiety | 35.5 (10.3) | 37.6 (11.0) | 0.52 |  | 34.4 (9.6) | 37.4 (13.2) | 0.68 |
|  |  |  |  |  |  |  |  |
| Cancer worry | 16.8 (5.3) | 17.3 (5.3) | 0.99 |  | 16.1 (5.9) | 17.5 (6.7) | 0.52 |

^c^Statistical differences between means were tested using the Mann-Whitney U test

SD=standard deviation

Supplementary Table S6. Predictors of health-related quality of life, anxiety and cancer worry at genetic testing in individuals with hereditary breast and ovarian cancer, Lynch syndrome and familial breast and colorectal cancer using linear regression

|  |  | Health related quality of life (Rand-36) | | | | | | |  | Anxiety (State-Trait Anxiety Inventory) | | | | | | |  | Cancer Worry (Cancer Worry Scale) | | |
| --- | --- | --- | --- | --- | --- | --- | --- | --- | --- | --- | --- | --- | --- | --- | --- | --- | --- | --- | --- | --- |
|  |  | Physical Component Score | | |  | Mental Component Score | | |  | Stait anxiety | | |  | Trait anxiety | | |  |  |  |  |
|  |  | Univariable | Multivariable  Model 1^a^ | Multivariable  Model 2^b^ |  | Univariable | Multivariable Model  1 ^a^ | Multivariable Model 2^b^ |  | Univariable | Multivariable Model  1 ^a^ | Multivariable Model 2^b^ |  | Univariable | Multivariable  Model  1 ^a^ | Multivariable  Model 2^b^ |  | Univariable | Multivariable Model  1 ^a^ | Multivariable  Model 2^b^ |
|  |  | β (95% CI) | β (95% CI) | β (95% CI) |  | β (95% CI) | β (95% CI) | β (95% CI) |  | β (95% CI) | β (95% CI) | β (95% CI) |  | β (95% CI) | β (95% CI) | β (95% CI) |  | β (95% CI) | β (95% CI) | β (95% CI) |
| Family diagnosis | HBOC | Ref |  | Ref |  | Ref |  | Ref |  | Ref |  | Ref |  | Ref |  | Ref |  | Ref |  | Ref |
|  | Lynch syndrome | 1.28 (-7.09 – 9.65) |  | -2.18 (-10.19 – 5.82) |  | 1.05 (-8.90 – 11.00) |  | -3.63 (-13.28 – 6.02) |  | -0.79 (-5.66 – 4.08) |  | 1.27 (-3.51 – 6.04) |  | -0.56 (-4.64 – 3.53) |  | 1.18 (-2.91 – 5.27) |  | -1.33 (-1.37 – 0.70) |  | -0.44 (-2.47 – 1.59) |
|  | Familial BC | -0.92 (-8.68 – 6.84) |  | 3.54 (-3.84 – 10.93) |  | -7.78 (-17.00 – 1.45) |  | -3.85 (-12.75 – 5.05) |  | 1.47 (-3.01 – 5.95) |  | -0.52 (-4.93 – 3.89) |  | 2.55 (-1.21 – 6.30) |  | 1.07 (-2.71 – 4.85) |  | 0.28 (-1.60 – 2.17) |  | -0.69 (-2.56 – 1.17) |
|  | Familial CRC | -2.10 (-13.71 – 9.51) |  | -7.92 (-19.42 –3.58) |  | -1.12 (-14.94 – 12.69) |  | -12.65 (-26.51 – 1.22) |  | -2.03 (-8.78 – 4.72) |  | 3.57 (-3.28 – 10.43) |  | -1.57 (-7.23 – 4.10) |  | 2.22 (-3.65 – 8.10) |  | -0.39 (-3.18 – 2.41) |  | 2.06 (-0.82 – 4.94) |
|  |  |  |  |  |  |  |  |  |  |  |  |  |  |  |  |  |  |  |  |  |
| Indication for testing | Predictive | Ref | Ref |  |  | Ref | Ref |  |  | Ref | Ref |  |  | Ref | Ref |  |  | Ref | Ref |  |
|  | Index case testing^c^ | **-18.65 (-24.67** – **-12.63)** | **-18.11 (-24.45 – -11.77)** |  |  | **-19.24 (-26.58** – **-11.90)** | **-15.65 (-23.13 – -8.16)** |  |  | -0.001 (-0.002 – 0.001) | -0.001 (-0.003 – 0.001) |  |  | -0.001 (-0.002 – 0.001) | -0.001 (-0.002 – 0.001) |  |  | **3.30 (1.79 – 4.80)** | **2.73 (1.17-4.28)** |  |
|  |  |  |  |  |  |  |  |  |  |  |  |  |  |  |  |  |  |  |  |  |
| Cancer diagnosis in the past year | No | Ref |  | Ref |  | Ref |  | Ref |  | Ref |  | Ref |  | Ref |  | Ref |  | Ref |  | Ref |
|  | Yes | **-17.68 (-23.10 – -12.26)** |  | **-18.22 (-24.11 – -12.33)** |  | **-16.56 (-23.25 – -9.87)** |  | **-12.05 (-19.15 – -4.95)** |  | **6.00 (2.67 – 9.32)** |  | 3.21 (-0.30 – -6.73) |  | **4.17 (1.35 – 6.99)** |  | 2.60 (-0.42 – 5.61) |  | **2.37 (0.98 – 3.77)** |  | **1.58 (0.10 – 3.07)** |
|  |  |  |  |  |  |  |  |  |  |  |  |  |  |  |  |  |  |  |  |  |
| Sex | Male | Ref | Ref | Ref |  | Ref | Ref | Ref |  | Ref | Ref | Ref |  | Ref | Ref | Ref |  | Ref | Ref | Ref |
|  | Female | **-10.75 (-17.53** – **-3.96)** | **-8.32 (-15.09 – -1.55)** | **-11.79 (-19.04 – -4.54)** |  | **-16.17 (-24.20** – **-8.15)** | **-11.68 (-19.68 – -3.68)** | **-14.71 (-23.45 – -5.98)** |  | **7.45 (3.53 – 11.37)** | **6.03 (2.07 – 10.00)** | **6.61 (2.29 – 10.93)** |  | **6.05 (2.75 – 9.36)** | **5.56 (2.17 – 8.95)** | **5.57 (1.87 – 9.27)** |  | **3.23 (1.61 – 4.85)** | **2.38 (0.72-4.05)** | **3.09 (1.27 – 4.91)** |
|  |  |  |  |  |  |  |  |  |  |  |  |  |  |  |  |  |  |  |  |  |
| Age | <40 | 1.34 (-8.14 – 10.81) | -1.77 (-10.80 – 7.26) | 0.78 (-8.08 – 9.63) |  | -4.17 (-15.11 – 6.78) | -6.53 (-17.20 – 4.14) | -3.82 (-14.49 – 6.85) |  | 4.20 (-1.03 – 9.44) | 3.85 (-1.44 – 9.14) | 3.87 (-1.42 – 9.15) |  | 3.68 (-0.83 – 8.19) | 3.31 (-1.22 – 7.83) | 3.20 (-1.33 – 7.73) |  | 1.47 (-0.79 – 3.73) | 1.57 (-0.67 – 3.82) | 1.47 (-0.78 – 3.72) |
|  | 40-60 | Ref | Ref | Ref |  | Ref | Ref | Ref |  | Ref | Ref | Ref |  | Ref | Ref | Ref |  | Ref | Ref | Ref |
|  | ≥61 | 4.83 (-1.41 – 11.08) | 1.39 (-4.85 – 7.63) | -1.79 (-8.22 – 4.65) |  | **14.06 (6.85 – 21.27)** | **9.53 (2.16** – **16.90)** | **8.42 (0.66 – 16.17)** |  | **-7.37 (-10.82 – -3.92)** | **-6.41 (-10.06 – -2.76)** | **-5.24 (-9.07 – -1.40)** |  | **-4.48 (-7.46 – -1.51)** | **-3.82 (-6.94 – -0.70)** | -3.00 (-6.29 – 0.29) |  | **-2.27 (-3.75 – -0.80)** | -1.38 (-2.93 – 0.17) | -1.26 (-2.88 – 0.36) |
|  |  |  |  |  |  |  |  |  |  |  |  |  |  |  |  |  |  |  |  |  |
| Education | Mandatory | 0.73 (-8.02 – 11.48) | -1.98 (-11.22 – 7.25) | -0.74 (-9.93 – 8.46) |  | 4.67 (-6.90 – 16.24) | -2.69 (-13.60 – 8.22) | -2.17 (-13.25 – 8.91) |  | -3.34 (-8.98 – 2.30) | 0.14 (-5.33 – 5.61) | 0.22 (-5.26 – 5.70) |  | -4.71 (-9.47 – 0.04) | -2.24 (-6.91 – 2.44) | -2.17 (-6.87 – 2.53) |  | -1.78 (-4.15 – 0.60) | -0.47 (-2.76 – 1.81) | -0.52 (-2.85 – 1.81) |
|  | High school | Ref | Ref | Ref |  | Ref | Ref | Ref |  | Ref | Ref | Ref |  | Ref | Ref | Ref |  | Ref | Ref | Ref |
|  | University | 2.36 (-5.42 – 10.14) | 5.32 (-1.94 – 12.58) | 5.46 (-1.82 – 12.75) |  | -3.89 (-13.12 – 5.34) | -0.45 (-9.02 – 8.13) | -0.43 (-9.21 – 8.35) |  | 1.46 (-3.04 – 5.96) | 0.34 (-3.97 – 4.64) | 0.27 (-4.08 – 4.61) |  | -2.48 (-2.67 – 1.32) | -3.31 (-6.99 – 0.37) | -3.57 (-7.29 – 0.15) |  | 0.08 (-1.81 – 1.97) | -0.64 (-2.43 – 1.16) | -0.37 (-2.22 – 1.47) |

^a^Variables included in the model: indication for testing, sex, age groups (<40, 40-60, ≥61) and education.

^b^Variables included in the model: family diagnosis, cancer diagnosis in the past year, sex, age groups (<40, 40-60, ≥61) and education.
BC=breast cancer, CI=Confidence interval, CRC=colorectal cancer, HBOC=hereditary breast and ovarian cancer, Ref=Reference

Supplementary Table S7. Predictors of health-related quality of life, anxiety and cancer worry 6 months after genetic testing in individuals with hereditary breast and ovarian cancer, Lynch syndrome and familial breast and colorectal cancer using linear regression

|  |  | Health related quality of life (Rand-36) | | | | | | |  | Anxiety (State-Trait Anxiety Inventory) | | | | | | |  | Cancer Worry (Cancer Worry Scale) | | |
| --- | --- | --- | --- | --- | --- | --- | --- | --- | --- | --- | --- | --- | --- | --- | --- | --- | --- | --- | --- | --- |
|  |  | Physical Component Score | | |  | Mental Component Score | | |  | Stait anxiety | | |  | Trait anxiety | | |  |  |  |  |
|  |  | Univariable | Multivariable  Model 1^a^ | Multivariable  Model 2^b^ |  | Univariable | Multivariable Model  1 ^a^ | Multivariable Model 2^b^ |  | Univariable | Multivariable Model  1 ^a^ | Multivariable Model 2^b^ |  | Univariable | Multivariable  Model  1 ^a^ | Multivariable  Model 2^b^ |  | Univariable | Multivariable Model  1 ^a^ | Multivariable  Model 2^b^ |
|  |  | β (95% CI) | β (95% CI) | β (95% CI) |  | β (95% CI) | β (95% CI) | β (95% CI) |  | β (95% CI) | β (95% CI) | β (95% CI) |  | β (95% CI) | β (95% CI) | β (95% CI) |  | β (95% CI) | β (95% CI) | β (95% CI) |
| Family diagnosis | HBOC | Ref |  | Ref |  | Ref |  | Ref |  | Ref |  | Ref |  | Ref |  | Ref |  | Ref |  | Ref |
|  | Lynch syndrome | 1.56 (-6.76 – 9.87) |  | -0.54 (-9.04 – 7.95) |  | -2.42 (-11.85 – 7.01) |  | -5.55 (-15.48 – 4.38) |  | 0.14 (-4.56 – 4.85) |  | 2.27 (-2.57 – 7.11) |  | -0.17 (-4.45 – 4.11) |  | 1.90 (-2.52 – 6.32) |  | -1.31 (-3.38 – 0.75) |  | -0.56 (-2.64 – 1.52) |
|  | Familial BC | 1.13 (-6.75 – 9.01) |  | 2.38 (-5.73 – 10.48) |  | -7.33 (-16.27 – 1.62) |  | -4.61 (-14.09 – 4.86) |  | 4.24 (-0.22 – 8.70) |  | 2.75 (-1.87 – 7.37) |  | **4.39 (0.33 – 8.45)** |  | 3.68 (-0.54 – 7.90) |  | 0.80 (-1.14 – 2.74) |  | 0.12 (-1.85 – 2.09) |
|  | Familial CRC | -0.76 (-12.16 – 10.64) |  | -4.67 (-16.93 – 7.59) |  | -5.34 (-18.27 – 7.60) |  | -10.64 (-24.97 – 3.69) |  | 0.60 (-7.01 – 5.90) |  | 3.26 (-3.72 – 10.24) |  | 0.09 (-5.79 – 5.96) |  | 2.90 (-3.48 – 9.28) |  | 0.42 (-2.41 – 3.25) |  | 2.44 (-0.56 – 5.45) |
|  |  |  |  |  |  |  |  |  |  |  |  |  |  |  |  |  |  |  |  |  |
| Indication for testing | Predictive | Ref | Ref |  |  | Ref | Ref |  |  | Ref | Ref |  |  | Ref | Ref |  |  | Ref | Ref |  |
|  | Index case testing | **-7.99 (-14.21 – -1.77)** | **-8.52 (-15.28 – -1.76)** |  |  | **-12.50 (-19.51 – -5.48)** | **-10.81 (-18.61 – -3.01)** |  |  | 0.00 (-0.002 – 0.002) | 0.00 (-0.002 – 0.002) |  |  | -0.001 (-0.002 – 0.001) | -0.001 (-0.002 – 0.001) |  |  | **3.21 (1.69 – 4.73)** | **2.62 (0.97 – 4.27)** |  |
|  |  |  |  |  |  |  |  |  |  |  |  |  |  |  |  |  |  |  |  |  |
| Cancer diagnosis in the past year | No | Ref |  | Ref |  | Ref |  | Ref |  | Ref |  | Ref |  | Ref |  | Ref |  | Ref |  | Ref |
|  | Yes | **-10.56 (-16.17 – -4.96)** |  | **-9.75 (-16.17 – -3.33)** |  | **-10.18 (-16.63 – -3.72)** |  | -5.77 (-13.28 – 1.74) |  | **6.23 (3.04 –** **9.41)** |  | **4.37 (0.71 – -8.02)** |  | **5.61 (2.70 – 8.53)** |  | **4.41 (1.07 – 7.75)** |  | **2.87 (1.48 – 4.26)** |  | **2.22 (0.65 – 3.79)** |
|  |  |  |  |  |  |  |  |  |  |  |  |  |  |  |  |  |  |  |  |  |
| Sex | Male | Ref | Ref | Ref |  | Ref | Ref | Ref |  | Ref | Ref | Ref |  | Ref | Ref | Ref |  | Ref | Ref | Ref |
|  | Female | **-7.85 (-14.68 – -1.03)** | -7.01 (-14.35 – 0.33) | **-9.32 (-17.04 – -1.60)** |  | **-9.88 (-17.65 – -2.10)** | -6.25 (-14.72 – 2.22) | **-9.58 (-18.60 –** **-0.55)** |  | **6.94 3.11 – 10.77)** | **6.11 (-1.98 – 10.24)** | **5.98 (1.58 – 10.37)** |  | **5.60 (2.08 – 9.11)** | **5.52 (1.72 – 9.32)** | **5.06 (1.04 – 9.08)** |  | **3.23 (1.56 – 4.90)** | **2.17 (0.38 – 3.95)** | **2.78 (0.89 – 4.68)** |
|  |  |  |  |  |  |  |  |  |  |  |  |  |  |  |  |  |  |  |  |  |
| Age | <40 | 8.95 (-1.11 – 19.01) | 8.14 (-2.12 – 18.40) | 8.84 (-1.20 – 18.86) |  | 5.17 (-6.26 – 16.60) | 3.30 (-8.54 – 15.13) | 5.26 (-6.47 – 16.99) |  | 1.23 (-4.33 – 6.78) | 0.37 (-5.31 – 6.04) | 1.05 (-4.56 – 6.67) |  | -0.83 (-5.96 – 4.31) | -1.79 (-7.01 – 3.44) | -1.25 (-6.38 – 3.88) |  | 1.22 (-1.23 – 3.66) | 1.25 (-1.26 – 3.75) | 1.33 (-1.13 – 3.79) |
|  | 40-60 | Ref | Ref | Ref |  | Ref | Ref | Ref |  | Ref | Ref | Ref |  | Ref | Ref | Ref |  | Ref | Ref | Ref |
|  | ≥61 | 4.27 (-1.88 – 10.43) | 3.88 (-2.65 – 10.41) | 1.39 (-5.52 – 8.30) |  | **8.57 (1.58 – 15.57)** | 4.93 (-2.61 – 12.46) | 4.95 (-3.13 – 13.03) |  | **-5.18 (-8.65 – 1.71)** | **-4.96 (-8.67 – -1.25)** | -3.57 (-7.51 – 0.37) |  | **-3.68 (-6.88 – -0.47)** | **-3.70 (-7.11 – -0.29)** | -2.36 (-5.96 – 1.24) |  | **-1.56 (-4.05 – -1.07)** | **-1.65 (-3.24 – -0.06)** | -1.22 (-2.91 – 0.48) |
|  |  |  |  |  |  |  |  |  |  |  |  |  |  |  |  |  |  |  |  |  |
| Education | Mandatory | 2.90 (-6.98 – 12.77) | 0.91 (-8.94 – 10.76) | 1.58 (-8.28 – 11.44) |  | 7.57 (-3.77 – 18.91) | 4.34 (-7.03 – 15.70) | 4.42 (-7.11 – 15.96) |  | -2.70 (-8.41 – 3.00) | -0.39 (-6.04 – 5.25) | -0.08 (-5.69 – 5.54) |  | -3.81 (-9.02 – 1.39) | -2.28 (-7.47 – 2.92) | -1.98 (-7.12 – 3.15) |  | -2.06 (-4.52 – 0.41) | -0.80 (-3.18 – 1.59) | -0.85 (-3.25 – 1.56) |
|  | High school | Ref | Ref | Ref |  | Ref | Ref | Ref |  | Ref | Ref | Ref |  | Ref | Ref | Ref |  | Ref | Ref | Ref |
|  | University | 4.59 (-3.14 – 12.32) | 7.00 (-0.64 – 14.63) | 7.36 (-0.38 – 15.09) |  | -0.84 (-9.72 – 8.04) | 1.62 (-7.19 – 10.43) | 1.47 (-7.58 – 10.51) |  | -0.60 (-5.07 – 3.87) | -1.92 (-6.30 – 2.46) | -2.45 (-6.86 –1.95) |  | -2.90 (-6.97 – 1.87) | -3.98 – (-8.01 – 0.05) | **-4.65 (-8.67 – -0.62)** |  | 0.75 (-1.19 – 2.70) | 0.05 (-1.82 – 1.91) | 0.15 (-1.75 – 2.05) |

^a^Variables included in the model: indication for testing, sex, age groups (<40, 40-60, ≥61) and education.

^b^Variables included in the model: family diagnosis, cancer diagnosis in the past year, sex, age groups (<40, 40-60, ≥61) and education.
BC=breast cancer, CI=Confidence interval, CRC=colorectal cancer, HBOC=hereditary breast and ovarian cancer, Ref=Reference

Supplementary Table S8. Quality of life measures, anxiety and cancer worry at baseline (0 months) and 6 months, stratified by sex, age group and education level compared to normative reference samples

|  | Sex | |  | Age groups | | | | | | | | | | |  | Education level^a^ | | |
| --- | --- | --- | --- | --- | --- | --- | --- | --- | --- | --- | --- | --- | --- | --- | --- | --- | --- | --- |
|  | Women | Men |  | ≤29 | 30-39 | 40-49 | 50-59 | 60-69 | 70-79 | ≥80 |  | ≤45 | 46-65 | ≥66 |  | Mandatory | High school/  Lower + Middle | University/  Higher |
| Physical functioning |  |  |  |  |  |  |  |  |  |  |  |  |  |  |  |  |  |  |
| 0 months | 74.6 (25.2) | 80.1 (21.1) |  | 85.0 (14.6) | 77.5 (30.7) | 79.9 (22.1) | 77.4 (24.5) | 72.4 (24.6) | 73.5 (23.1) | 71.3 (30.0) |  |  |  |  |  | 67.3 (26.9) | 74.1 (24.5) | 78.7 (23.5) |
| 6 months | 79.5 (21.3) | 84.1 (16.8) |  | 97.5 (4.2) | 90.2 (13.2) | 85.1 (17.8) | 81.3 (20.7) | 76.2 (22.4) | 74.9 (20.6) | 76.3 (14.4) |  |  |  |  |  | 71.8 (20.6) | 78.5 (23.3) | 83.3 (18.9) |
| Norm population | 81.4 (26.0) | 85.6 (21.5) |  | 91.7 (14.3) | 91.1 (16.5) | 88.9 (16.3) | 85.1 (18.7) | 80.3 (24.3) | 72.6 (33.2) | 49.7 (43.2) |  |  |  |  |  | 68.4 (35.1) | 84.4 (22.0) | 89.7 (17.4) |
| Role functioning/ physical |  |  |  |  |  |  |  |  |  |  |  |  |  |  |  |  |  |  |
| 0 months | 44.7 (43.8) | 65.9 (42.9) |  | 40.6 (46.2) | 46.3 (48.1) | 36.0 (43.2) | 48.1 (44.7) | 52.2 (44.5) | 60.4 (42.4) | 74.0 (33.6) |  |  |  |  |  | 50.7 (45.0) | 51.4 (45.1) | 48.4 (44.6) |
| 6 months | 56.7 (42.2) | 67.5 (39.1) |  | 83.3 (25.8) | 61.8 (47.4) | 60.0 (43.4) | 53.3 (42.9) | 62.5 (41.6) | 56.3 (38.0) | 75.0 (35.4) |  |  |  |  |  | 53.6 (43.4) | 56.9 (42.0) | 61.2 (40.9) |
| Norm population | 73.1 (40.8) | 77.8 (34.3) |  | 83.3 (25.1) | 81.8 (31.0) | 79.6 (28.7) | 77.3 (31.7) | 75.8 (40.4) | 65.2 (53.2) | 36.4 (64.7) |  |  |  |  |  | 59.3 (52.2) | 76.7 (34.9) | 81.1 (32.5) |
| Role functioning/ emotional |  |  |  |  |  |  |  |  |  |  |  |  |  |  |  |  |  |  |
| 0 months | 58.3 (44.1) | 83.2 (31.9) |  | 38.5 (38.6) | 57.1 (43.6) | 52.9 (46.5) | 57.4 (45.5) | 71.3 (38.8) | 77.8 (38.2) | 95.8 (10.2) |  |  |  |  |  | 68.9 (41.0) | 67.6 (41.6) | 60.4 (43.9) |
| 6 months | 64.5 (41.3) | 80.5 (34.2) |  | 55.6 (50.2) | 71.9 (38.9) | 63.8 (43.1) | 63.9 (42.7) | 70.6 (38.9) | 75.9 (37.0) | 75.0 (16.7) |  |  |  |  |  | 77.1 (35.9) | 70.8 (40.1) | 65.1 (41.3) |
| Norm population | 73.4 (39.8) | 79.9 (32.8) |  | 76.6 (29.1) | 75.0 (35.0) | 77.6 (29.6) | 78.8 (30.7) | 82.4 (35.3) | 77.4 (46.5) | 57.3 (64.8) |  |  |  |  |  | 70.7 (47.4) | 78.4 (33.7) | 77.2 (34.7) |
| Energy |  |  |  |  |  |  |  |  |  |  |  |  |  |  |  |  |  |  |
| 0 months | 51.7 (23.2) | 63.0 (22.7) |  | 34.4 (15.7) | 51.3 (24.6) | 50.3 (27.3) | 47.7 (23.5) | 56.8 (20.5) | 69.9 (17.8) | 67.5 (10.4) |  |  |  |  |  | 60.24 (24.3) | 52.7 (24.9) | 52.9 (22.8) |
| 6 months | 55.5 (23.7) | 68.7 (21.8) |  | 47.5 (18.6) | 53.2 (24.7) | 54.4 (25.7) | 52.5 (25.1) | 61.9 (21.5) | 70.5 (19.4) | 78.8 (14.4) |  |  |  |  |  | 66.0 (24.1) | 55.9 (25.3) | 58.3 (22.9) |
| Norm population | 58.5 (23.7) | 63.6 (20.4) |  | 58.9 (16.8) | 57.7 (19.4) | 59.4 (17.6) | 61.4 (20.3) | 66.6 (23.8) | 66.8 (28.6) | 55.4 (35.3) |  |  |  |  |  | 60.1 (29.0) | 60.9 (21.2) | 61.7 (20.2) |
| Emotional wellbeing |  |  |  |  |  |  |  |  |  |  |  |  |  |  |  |  |  |  |
| 0 months | 68.5 (20.4) | 78.8 (17.7) |  | 53.5 (20.8) | 61.7 (19.6) | 66.8 (19.8) | 66.4 (19.7) | 75.1 (18.6) | 82.9 (17.6) | 78.7 (19.4) |  |  |  |  |  | 76.8 (18.9) | 69.6 (20.9) | 68.9 (20.3) |
| 6 months | 72.1 (18.5) | 80.9 (18.3) |  | 66.0 (12.3) | 74.1 (14.5) | 69.8 (18.4) | 71.1 (19.4) | 74.3 (19.7) | 83.4 (17.1) | 88.0 (12.6) |  |  |  |  |  | 80.4 (18.3) | 70.6 (21.0) | 73.4 (18.4) |
| Norm population | 74.1 (19.8) | 78.1 (17.3) |  | 73.2 (14.4) | 74.8 (16.1) | 75.6 (14.2) | 76.6 (17.5) | 79.5 (20.2) | 79.5 (24.3) | 73.3 (30.8) |  |  |  |  |  | 75.8 (25.3) | 76.1 (18.0) | 76.3 (16.4) |
| Social functioning |  |  |  |  |  |  |  |  |  |  |  |  |  |  |  |  |  |  |
| 0 months | 78.0 (24.9) | 64.7 (30.0) |  | 50.0 (29.1) | 65.2 (29.5) | 65.1 (23.4) | 61.2 (33.7) | 71.6 (27.0) | 76.7 (27.9) | 91.7 (10.2) |  |  |  |  |  | 74.4 (27.6) | 68.8 (28.6) | 65.0 (30.0) |
| 6 months | 73.4 (25.1) | 80.4 (25.3) |  | 72.9 (25.5) | 75.0 (22.8) | 73.9 (27.2) | 70.5 (26.0) | 74.0 (26.6) | 84.4 (20.6) | 90.6 (12.0) |  |  |  |  |  | 80.4 (24.5) | 73.8 (27.0) | 74.2 (25.3) |
| Norm population | 80.8 (25.3) | 84.8 (21.4) |  | 83.5 (17.3) | 82.9 (21.1) | 83.5 (18.7) | 82.1 (21.6) | 85.7 (23.6) | 83.6 (30.1) | 72.4 (42.6) |  |  |  |  |  | 78.5 (31.9) | 83.2 (22.1) | 84.2 (21.0) |
| Pain |  |  |  |  |  |  |  |  |  |  |  |  |  |  |  |  |  |  |
| 0 months | 66.5 (27.6) | 77.8 (23.7) |  | 59.1 (27.5) | 76.8 (32.1) | 70.5 (25.8) | 68.3 (27.8) | 64.9 (27.2) | 74.8 (23.5) | 70.8 (27.2) |  |  |  |  |  | 69.9 (27.9) | 63.4 (29.7) | 70.4 (26.2) |
| 6 months | 71.4 (25.8) | 75.5 (25.3) |  | 85.4 (9.4) | 78.2 (23.4) | 72.4 (27.2) | 69.0 (27.2) | 68.7 (27.4) | 77.5 (21.0) | 86.3 (16.4) |  |  |  |  |  | 72.7 (25.6) | 67.4 (28.7) | 73.7 (24.9) |
| Norm population | 73.1 (27.5) | 76.3 (23.9) |  | 82.2 (18.1) | 80.1 (21.9) | 76.2 (20.5) | 71.3 (22.9) | 71.8 (27.9) | 69.8 (34.3) | 60.0 (41.8) |  |  |  |  |  | 66.2 (34.2) | 73.7 (24.8) | 80.1 (21.7) |
| General health |  |  |  |  |  |  |  |  |  |  |  |  |  |  |  |  |  |  |
| 0 months | 59.7 (20.3) | 64.0 (21.7) |  | 55.0 (15.8) | 64.2 (23.5) | 61.5 (22.5) | 56.2 (20.7) | 60.9 (21.0) | 66.1 (17.1) | 64.2 (18.0) |  |  |  |  |  | 60.6 (19.9) | 58.6 (21.8) | 61.5 (20.7) |
| 6 months | 62.4 (21.3) | 68.6 (18.3) |  | 60.0 (15.8) | 64.7 (20.8) | 66.7 (21.0) | 60.0 (20.6) | 62.4 (23.7) | 69.9 (13.9) | 68.8 (27.8) |  |  |  |  |  | 68.3 (19.6) | 59.3 (22.7) | 64.5 (20.6) |
| Norm population | 66.9 (23.5) | 69.3 (19.4) |  | 71.7 (16.8) | 71.5 (19.3) | 70.6 (16.8) | 67.0 (19.1) | 67.0 (22.8) | 64.5 (27.0) | 53.7 (31.2) |  |  |  |  |  | 59.4 (26.6) | 68.8 (20.3) | 71.2 (19.6) |
| Stait anxiety |  |  |  |  |  |  |  |  |  |  |  |  |  |  |  |  |  |  |
| 0 months |  |  |  |  |  |  |  |  |  |  |  | 47.0 (12.9) (Women)  39.2 (18.4) (Men) | 41.0 (13.9) (Women)  35.7 (10.4) (Men) | 35.1 (10.3) (Women)  30.9 (9.3) (Men) |  |  |  |  |
| 6 months |  |  |  |  |  |  |  |  |  |  |  | 42.4 (12.3) (Women)  31.6 (5.9) (Men) | 38.5 (12.7) (Women)  34.7 (12.1) (Men) | 34.6 (10.7) (Women)  29.2 (9.6) (Men) |  |  |  |  |
| Norm population |  |  |  |  |  |  |  |  |  |  |  | 33.8 (9.9) (Women)  35.0 (10.6) (Men) | 36.3 (11.0) (Women)  30.8 (8.0) (Men) | 33.0 (8.4) (Women)  31.0 (8.5) (Men) |  |  |  |  |
| Cancer worry |  |  |  |  |  |  |  |  |  |  |  |  |  |  |  |  |  |  |
| 0 months | 18.0 (5.6) | 14.8 (4.4) |  | 20.4 (6.8) | 19.3 (5.7) | 18.4 (6.6) | 18.0 (5.7) | 16.7 (5.1) | 14.4 (3.5) | 15.5 (3.0) |  |  |  |  |  | 15.8 (4.7) | 17.5 (5.8) | 17.6 (5.6) |
| 6 months | 17.4 (5.3) | 14.2 (5.1) |  | 20.3 (5.3) | 19.2 (5.7) | 17.8 (5.3) | 17.4 (5.8) | 16.2 (5.0) | 13.4 (4.1) | 13.3 (3.1) |  |  |  |  |  | 14.4 (4.8) | 16.5 (5.9) | 17.2 (5.5) |
| Norm population | 14.0 (4.1) | 12.6 (3.5) |  | 12.8 (3.7) | 13.7 (4.4) | 13.3 (4.2) | 13.5 (3.8) | 13.2 (3.6) | 13.1 (3.5) |  |  |  |  |  |  | 13.3 (3.9) | 13.6 (4.0) | 12.9 (3.6) |

^a^Education in the study sample and in Ohlsson-Nevo et al. is categorized as mandatory (grade 0–9), high school (grade 10–12) and university education (>12 years), whereas in Hawranek et al., education is categorized into lower (high school or less), middle (up to 2 years at post-secondary level) and higher (over 2 years at post-secondary level).

^b^The oldest age group in Hawranek et al. includes participants aged 70-74 years.
